# CRISPR-Cas genome engineering of esterase activity in *Saccharomyces cerevisiae* steers aroma formation

**DOI:** 10.1186/s13104-018-3788-5

**Published:** 2018-09-27

**Authors:** Alexander Dank, Eddy J. Smid, Richard A. Notebaart

**Affiliations:** Food Microbiology, Wageningen University & Research, P.O. Box 17, 6700 AA Wageningen, The Netherlands

**Keywords:** *Saccharomyces cerevisiae*, Esterase, Aroma, CRISRP-Cas9

## Abstract

**Objective:**

*Saccharomyces cerevisiae* is used worldwide for the production of ale-type beers. This yeast is responsible for the production of the characteristic fruity aroma compounds. Esters constitute an important group of aroma active secondary metabolites produced by *S. cerevisiae.* Previous work suggests that esterase activity, which results in ester degradation, may be the key factor determining the abundance of fruity aroma compounds. Here, we test this hypothesis by deletion of two *S. cerevisiae* esterases, *IAH1* and *TIP1*, using CRISPR-Cas9 genome editing and by studying the effect of these deletions on esterase activity and extracellular ester pools.

**Results:**

*Saccharomyces cerevisiae* mutants were constructed lacking esterase *IAH1* and/or *TIP1* using CRISPR-Cas9 genome editing. Esterase activity using 5-(6)-carboxyfluorescein diacetate (cFDA) as substrate was found to be significantly lower for Δ*IAH1* and Δ*IAH1ΔTIP1* mutants compared to wild type (WT) activity (P < 0.05 and P < 0.001, respectively). As expected, we observed an increase in relative abundance of acetate and ethyl esters and an increase in ethyl esters in Δ*IAH1* and Δ*TIP1*, respectively. Interestingly, the double gene disruption mutant Δ*IAH1ΔTIP1* showed an aroma profile comparable to WT levels, suggesting the existence and activation of a complex regulatory mechanism to compensate multiple genomic alterations in aroma metabolism.

**Electronic supplementary material:**

The online version of this article (10.1186/s13104-018-3788-5) contains supplementary material, which is available to authorized users.

## Introduction

*Saccharomyces cerevisiae* is a fermentative eukaryotic microorganism that is extensively used in the beer brewing industry. Currently, the demand for new types of beer is growing [1] which increases the interest in methods to steer the production of aroma compounds by yeast cultures during fermentation.

Recently, van Rijswijck et al. [2] showed that *Cyberlindnera fabianii* displays low esterase activity and at the same time produces a high abundance of aroma compounds compared to *Pichia kudriavzevii* and *S. cerevisiae* which display high esterase activity and relatively low production of aroma active esters. A high esterase activity therefore may be the limiting factor for the overall abundance of esters and thus a key factor in determining aroma profiles of fermented alcoholic beverages. Here, we test this hypothesis by deletion of genes encoding esterases from the genome of *S. cerevisiae*.

An important acetate ester, isoamyl acetate, is hydrolysed by Iah1p, which is encoded by *IAH1* [3]. Mutants lacking Iah1p showed an increase in isoamyl acetate, ethyl acetate and isobutyl acetate, which may suggest a reduction of hydrolysis of several acetate esters. *TIP1* encodes for a protein with putative esterase activity towards esters of fatty acid chains ranging from 4 to 16 carbon atoms [4]. Therefore a knock-out of this gene may result in an increase of esters derived from free fatty acids and ethanol, like ethyl hexanoate and ethyl octanoate. In this study *S. cerevisiae* BY4742 mutants lacking *IAH1* and *TIP1* were constructed using CRISPR-Cas9. Esterase activity was quantified using a fluorescent acetate ester as substrate and aroma production was monitored by GC–MS. The relationship between esterase activity and aroma production was investigated for wild type (WT) and mutant strains.

## Main text

### Methods

#### Strain and media

*Saccharomyces cerevisiae* BY4742 [5] was grown in synthetic complete (SC) medium with individual amino acid supplementation according to selective requirements (see Additional file 1). *S. cerevisiae* BY4742 and mutant thereof were grown on malt extract agar (MEA) and malt extract broth (MEB) supplemented with amino acids according to the levels in SC media and 50 g/L glucose during cultivation.

#### Guide RNA plasmid construction

Guide RNA (gRNA) sequences targeting *IAH1* and *TIP1* were designed using CHOPCHOP [6]. These gRNAs, which are predicted to have no off-targets, were constructed by PCR on p426-SNR52p-gRNA.CAN1/Y-SUP4t plasmids (Addgene plasmid #43803 [7]) using forward (FW) primers with 20 nucleotide guide sequence overhang. The linearized plasmid fragments were blunt-end ligated into plasmids and propagated in competent *Escherichia coli* dh5α cells (Thermofisher Scientific, The Netherlands) (see Additional file 2). Constructed plasmids were sequenced for validation of correct guide RNA integration (Additional file 3).

#### Gene disruption

p415-GaIL-TEF1-CAS9-CYC1t (Addgene plasmid #43804 [7], modified in-house via homologous recombination of TEF1 promotor between GaIL and CAS9 in plasmid p415-GaIL-CAS9-CYC1t), p426-SNR52p-gRNA.*IAH1*/Y-SUP4t or p426-SNR52p-gRNA.*TIP1*/Y-SUP4t (this study) and linear DNA repair templates (see Additional file 4) were transferred into *S. cerevisiae* BY4742 by electroporation according to an adapted method of DiCarlo et al. [7] described in Additional file 5.

Genomic DNA was extracted according to the method of Lõoke et al. [8]. Gene deletion was confirmed by PCR and by Sanger sequencing (Baseclear, The Netherlands) (see Additional file 6).

Δ*IAH1* mutant was cured of p426-SNR52p-gRNA.IAH1/Y SUP4t using 750 μg/mL 5-fluoroorotic acid in SC plates. The electroporation protocol was repeated for the cured ΔIAH1 mutant using p426-SNR52p-gRNA.TIP1/Y SUP4t for construction of ΔIAH1ΔTIP1 double mutant.

#### Culture conditions

Fermentation was performed according to the method of van Rijswijck et al. [2] (see Additional file 7). 2 mL samples were stored at − 20 °C until analysis and the rest of the cells were collected for cell free extract preparation.

#### Esterase activity

Cell free extracts were prepared and esterase activity was determined according to the method of van Rijswijck et al. [2] (see Additional file 7).

#### Volatile organic compounds (VOCs) analysis

VOCs in each sample were determined by headspace solid-phase microextraction gas chromatography mass spectrometry (HS-SPME GC–MS) (see Additional file 7). Relative abundancy was calculated by the formula used by van Rijswijck et al. [2]:

$$Relative abundance \left( y \right) of compound \left( x \right) = \log_{2} \left( {\frac{{MSquantitation\left( {xy} \right)}}{{Median\left( {MSquantitation\left( x \right)} \right)}}} \right)$$

Heat maps were constructed using relative abundancy data in JMP 13 statistical software (SAS, USA).

#### Statistical analysis

All statistical data analysis was performed by JMP 13 statistical software (SAS, USA).

### Results

#### Esterase activity

**Fig. 1:**
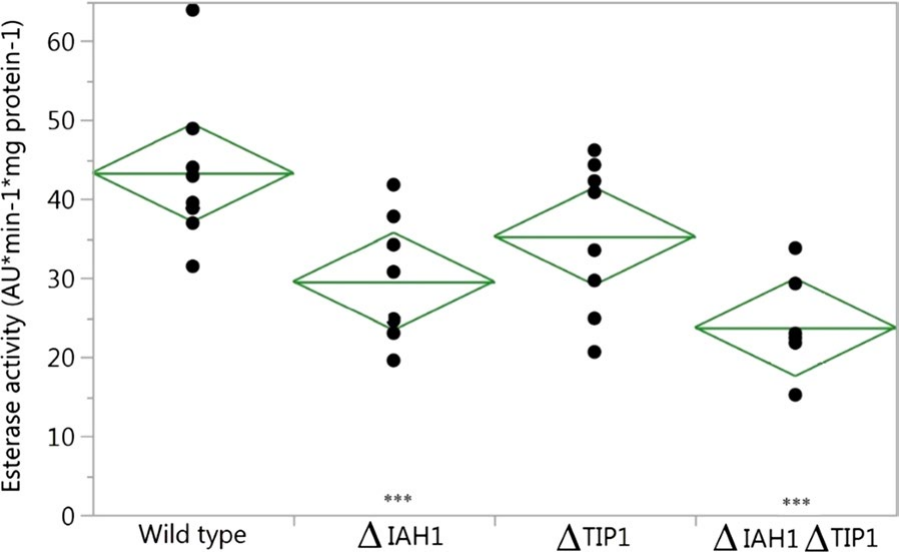
Specific cFDA hydrolysing activity in AU min^−1^ mg protein^−1^ (n = 8) of cell free extracts from cultures of WT, *ΔIAH1*, *ΔTIP1* and *ΔIAH1TIP1*. Diamonds represent 95% confidence interval (upper and lower tip) with mean represented by the middle line. *** Indicate significant difference between hydrolysing activity with WT (P < 0.001 for *ΔIAH1ΔTIP1* and P < 0.05 for *ΔIAH1*)

Ester hydrolysing activity was determined by monitoring the increase of fluorescence by enzymatic hydrolysis of 5-(and 6)-carboxyfluorescein diacetate (cFDA) to 5-(and 6)-carboxyfluorescein (cF). Hydrolysis of the non-fluorescent cFDA to fluorescent cF was recorded real-time as the increase of fluorescence which is correlated to the amount of substrate hydrolysed [expressed as arbitrary units (AU)]. The hydrolysis rate was expressed as amount of substrate converted in arbitrary units per minute per mg cell protein, as shown in Fig. 1.

The average hydrolysis rate found for the WT is 43.3 ± 9.8 AU min^−1^ mg protein^−1^. Mutant strains Δ*IAH1*, Δ*TIP1* and Δ*IAH1ΔTIP1* showed hydrolysis rates of 29.9 ± 7.9, 35.3 ± 9.5 and 23.7 ± 5.6 AU min^−1^ mg protein^−1^, respectively, where Δ*IAH1ΔTIP1* (P < 0.001) and Δ*IAH1* (P < 0.05) are significantly different from WT.

#### Distinct aroma profiles

The VOCs present in each sample were determined using HS SPME GC–MS after static incubation on MEB with 50 g/L glucose for 48 h at 30 °C.

Six groups of compounds based on their chemical characteristics were distinguished in the aroma profiles of all strains; ethanol, higher alcohols, ethyl esters, acetate esters, ethyl acetate and fatty acids.

**Fig. 2:**
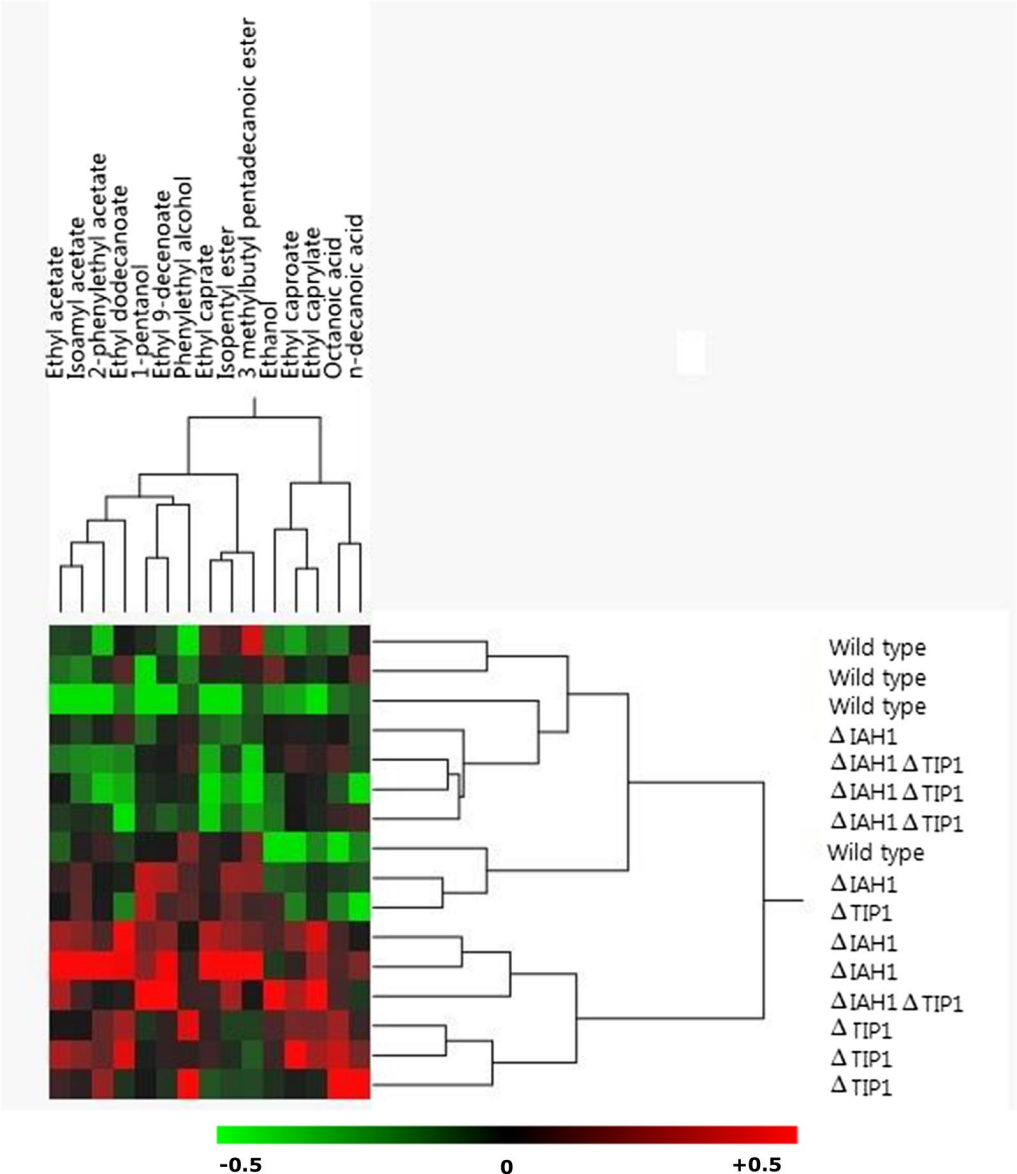
Heat map of correlation for relative abundance for volatile organic compounds produced by WT, *ΔIAH1*, *ΔTIP1 and ΔIAH1ΔTIP1* mutant strains. Green = below average abundancy (− 0.5), black = average abundancy (0), red = above average abundancy (+ 0.5)

Hierarchical clustering of the strains yielded three main clusters (Fig. 2), (i) one cluster containing relatively low abundancy of aroma consisting of three WT samples, three Δ*IAH1ΔTIP1* double mutant replicates and one Δ*IAH1* mutant replicate, (ii) an intermediate cluster containing one WT, one Δ*IAH1* mutant and one Δ*TIP1* mutant and (iii) one cluster containing relatively high abundancy of aroma consisting of three single Δ*TIP1* mutant replicates, two Δ*IAH1* mutant replicates and one Δ*IAH1ΔTIP1* double mutant replicate. Hierarchical clustering of compounds resulted in a cluster of acetate esters (left), fatty acids (right) and several medium-chain fatty acid (MCFA) ethyl esters. The clustering of different replicates and of compounds hydrolysed by *IAH1* and *TIP1* show that the deletion of esterases *IAH1* and *TIP1* induces qualitative changes in the profile of aroma compounds secreted by *S. cerevisiae*.

## Discussion

Esters are important compounds for the flavour characteristics in fermented products. Esters can be divided into two groups; acetate esters and ethyl esters. Most acetate esters are derived from higher alcohols originating from amino acid catabolism that combine with acetyl-CoA through a condensation reaction catalysed by alcohol-*O*-acetyl transferases [9]. Production of ethyl esters from ethanol and acyl-CoA, derived from free fatty acids, is catalysed by acyl-CoA:ethanol *O*-acyltransferases [10]. However, next to formation of esters, also the availability of precursors and ester hydrolysing activity are factors determining aroma profiles [2].

In this study the relationship between esterase activity and the production of volatile organic compounds was investigated. Knock-out strains with deletions of esterase encoding genes Δ*IAH1*, Δ*TIP1* and Δ*IAH1*Δ*TIP1* were constructed using CRISPR-Cas9. Compared to the WT strain, the Δ*IAH1* and Δ*IAH1*Δ*TIP1* deletion mutants showed a significant reduction of the total ester hydrolysis activity using cFDA as substrate (69% and 55% respectively). These findings suggest that the esterase encoded by the gene *IAH1* strongly contributes to the hydrolysis of cFDA. The mutant lacking gene *TIP1* (Δ*TIP1*) showed a reduced, albeit not significant, esterase activity compared to WT. Possibly, Tip1p has low affinity for cFDA as it has been described to hydrolyse fatty acid ethyl esters with a carbon size ranging from 4 to 16 [4], limiting Tip1p contribution towards acetate ester hydrolysis. It may also be possible that other esterases encoded on the genome partially compensate the loss of *TIP1*. Therefore, future research needs to be focused on multiplex CRISPR-Cas genome engineering to systematically knockout arrays of esterases.

Hierarchical clustering of the relative abundancy of aroma compounds showed that replicate samples of WT, Δ*IAH1*, Δ*TIP1* and Δ*IAH1*Δ*TIP1* cluster together. Furthermore, clustering of compounds revealed acetate esters to cluster together, which suggests that the deletion of *IAH1* indeed affects the relative abundance of compounds belonging to this group. Ethyl acetate, isoamyl acetate and 2-phenylethyl acetate indeed were shown to be relatively higher in abundance in Δ*IAH1* compared to the WT, in line with findings of Fukuda et al. [11] and Fukuda et al. [12]. Interestingly, Δ*IAH1* also showed an increase of several MCFA ethyl esters compared to the WT. Many enzymes in metabolism have promiscuous activities for a range of different substrates [13]. As such, it may be possible that Iah1p has promiscuous activity for MCFA ethyl esters. Alternatively the absence of esterase activity linked to Iah1p may result in redirection of intracellular fluxes of amino acid catabolism intermediates, like α-keto-isocaproate and isovaleryl-CoA, towards MCFA ethyl esters [14].

Mutant Δ*TIP1* showed an increase of relative abundance of ethyl acetate, 2-phenylethyl acetate, phenylethyl alcohol, ethyl caproate, ethyl caprylate and fatty acids. Although esterase activity measured as hydrolysis of cFDA was found to be not significantly different between Δ*TIP1* and the WT, hierarchal clustering does show altered aroma profiles for Δ*TIP1* replicates compared to the WT aroma profiles. Interestingly, 3 out of 4 Δ*IAH1ΔTIP1* replicates were found to cluster with three WT replicates, indicating that Δ*IAH1ΔTIP1* double mutant and the WT produce similar aroma profiles. Although esterase activity on cFDA was found to be significantly lower for Δ*IAH1ΔTIP1* compared the WT (23.7 ± 5.6 versus 43.3 ± 9.8 AU min^−1^ mg protein^−1^ respectively), no large shifts in aroma profiles were detected. Disruption of both *IAH1* and *TIP1* did thus not result in accumulation of effects of both single deletion, but rather caused an effect resulting in WT phenotypic behaviour. This strongly suggests that deletion of both esterases resulted in changes within the metabolism of the cell leading to altered ester production compared to single mutant strains. It is proposed that deletion of both *IAH1* and *TIP1* resulted in a regulatory response in the cells leading to either rewiring of fluxes, thereby decreasing ester substrates, decrease of ester synthase activity to compensate for the reduced hydrolysing activity or up-regulation of promiscuous esterases.

Analysing the substrate pool for ester production and monitoring expression levels of key genes involved in ester formation (ea. *ATF1*, *ATF2*, *lg*-*ATF1* [9] and *EHT1*, *EEB1* [10]) may further elucidate the mechanisms important in regulating aroma production in *S. cerevisiae.*

To conclude, in this study single and double gene knockouts of *IAH1* and *TIP1* were constructed using CRISPR-Cas (i.e., Δ*IAH1*, Δ*TIP1* and Δ*IAH1*Δ*TIP1*). Guide RNA vectors targeting *IAH1* and *TIP1* were constructed and transferred into *S. cerevisiae* in combination with donor DNA templates causing effective deletion of the target gene through homologous recombination.

Esterase activity and aroma profiles were notably affected by these deletions. The results of this study therefore further advance a previous study by van Rijswijck et al. [2] suggesting a crucial link between esterase activity and aroma production during fermentation. Moreover, our findings also suggest that ester production may be strictly regulated in *S. cerevisiae,* thereby limiting the effect of esterase disruption. Disruption of esterases may alter intercellular balances of metabolites causing a regulatory response. Identifying such regulatory systems for aroma production may lead to the ability to adapt aroma formation during fermentation by genetic engineering strategies.

## Limitations

- The enzyme assay test results for esterase activity on cFDA are dependent on the affinity of esterases to the substrate. The use of cFDA biases esterase activity to one substrate, whilst affinity per (acetate) ester may differ.
- Affinity of Tip1p for cFDA used in the enzyme assay may be low, which led to undetectable changes in esterase activity for esterases.
- This study focusses on two esterases. However multiple esterases are encoded in the genome that could affect esterase activity and consequently aroma formation. In the future, multiplex genome engineering is required to systematically knockout all esterases, thereby fully elucidating the link between esterase activity and aroma production.

## Additional files

**Additional file 1.** Media composition of synthetic complete.

**Additional file 2.** Description of all methods used for guide RNA construction.

**Additional file 3.** Confirmation results for guide RNA construction

**Additional file 4.** DNA repair fragment sequences used in this study during transformations.

**Additional file 5.** Protocol used to transform linear repair fragment DNA and plasmids into S*. cerevisiae*

**Additional file 6.** Sequencing result confirming gene deletion of *IAH1* and *TIP1* in mutant strains.

**Additional file 7.** Protocols used to cultivate *S. cerevisiae*, to monitor esterase activity and to obtain aroma profiles.

## Abbreviations

AU: arbitrary units
cF: 5-(6)-carboxyfluorescein
cFDA: 5-(6)-carboxyfluorescein diacetate
FW: forward
MCFA: medium-chain fatty acid
MEA: malt extract agar
MEB: malt extract broth
PCR: polymerase chain reaction
RV: reverse
SC: synthetic complete
VOC: volatile organic compounds
