# Supplementary material for: CRISPR-Cas genome engineering of esterase activity in Saccharomyces cerevisiae steers aroma formation
Source: BMC Res Notes. 2018 Sep 27;11:682. doi: 10.1186/s13104-018-3788-5 (PMC6161353; doi:10.1186/s13104-018-3788-5)
Supplement: Supplementary file 1 — Additional file 1. Media composition of synthetic complete. [file 13104_2018_3788_MOESM1_ESM.docx]

**Additional file 1: Synthetic complete media**

SC media composition is shown in the table below. SC media was filter sterilised using 0.2 µm filters.

| **Component** | **Gram/L** |
| --- | --- |
| Yeast nitrogen base | 1.7 |
| (NH_4_)_2_SO_4_ | 6 |
| L-histidine | 0.125 |
| L-Leucine* | 0.5 |
| Lysine monohydrocholoride | 0.1 |
| L-uracil** | 0.15 |
| Glucose | 20 |
| (Agar***) | 15 |

*L-leucine was only added when selecting for plasmid p415-GaIL-TEF1-CAS9-CYC1t

** L-uracil was only added when selecting for plasmid p426-SNR52p-gRNA.TIP1/Y-SUP4t

*** Agar was only added for SC plates. Agar was autoclaved separately, cooled to 50°C and added to filter sterilised media.
