# Supplementary material for: CRISPR-Cas genome engineering of esterase activity in Saccharomyces cerevisiae steers aroma formation
Source: BMC Res Notes. 2018 Sep 27;11:682. doi: 10.1186/s13104-018-3788-5 (PMC6161353; doi:10.1186/s13104-018-3788-5)
Supplement: Supplementary file 2 — Additional file 2. Description of all methods used for guide RNA construction. [file 13104_2018_3788_MOESM2_ESM.docx]

**Additional file 2: Methods used for guide RNA plasmid construction**

Guide RNA sequences targeting *IAH1* and *TIP1* were cloned into p426-SNR52p-gRNA.CAN1/Y-SUP4t plasmids (Addgene plasmid #43803 [6]) using polymerase chain reaction (PCR). Linear fragments containing the guide sequence were created with PCR using forward (FW) primers with 20 nucleotide guide sequence overhang. The linearized plasmid fragments containing the guide sequence were blunt-end ligated into plasmids and propagated in competent *Escherichia coli* dh5α cells (Thermofisher Scientific, The Netherlands).

FW primer 5’-ATTATCGATAAATTCGGGGAGTTTTAGAGCTAGAAATAGCAAGTTAAAATAAGGC-3’ and phosphorylated P426 reverse (RV) primer 5’ (P)-GATCATTTATCTTTCACTGCGGAGAAG-3’ were used for cloning the *IAH1* guide sequence.

FW primer 5’-GGAAGAAGCTGGGGAAACGGGTTTTAGAGCTAGAAATAGCAAGTTAAAATAAGGC-3’ and universal P426 reverse(RV) primer were used for cloning *TIP1* guide sequence.

Fifty ng PCR product was ligated using 7.5 Weiss U T4 DNA ligase (Thermofisher Scientific, The Netherlands), 1 μl 10X T4 ligase buffer (Thermofisher Scientific, The Netherlands) and MilliQ water to an end volume of 10 μl. Ligation mixtures were incubated 16h at 22°C.

Ligation products were transformed into competent *E. coli* DH5α cells (Thermofisher Scientific, The Netherlands) according to the following protocol:

1. Thaw 200 µL competent E. coli DH5a cells on ice for 10 min
2. Transfer 70 µL thawed E. coli DH5a cells to 15 mL tube
3. Add approximately 50 ng ligated PCR product and mix by tapping
4. Incubate on ice for 30 minutes (Mix by tapping and) heat shock at 42 °C for 45 seconds
5. Incubate on ice for 5 - 10 minutes
6. Add 0.9 ml SOC medium
7. Incubate 2h at 37 °C, 200 RPM
8. Plate out 50 μl, 200 μl on LB plates containing 50 µg/mL ampicillin
9. Spin down remaining culture, resuspend in 200 μl SOC and plate on LB plates containing 50 µg/mL ampicillin

3 colonies per transformation were picked and grown overnight in 5 mL liquid LB containing 50 µg/mL ampicillin. Plasmids were isolated from the overnight culture using QIAprep miniprep kit (Qiagen, Germany).
