# Supplementary material for: CRISPR-Cas genome engineering of esterase activity in Saccharomyces cerevisiae steers aroma formation
Source: BMC Res Notes. 2018 Sep 27;11:682. doi: 10.1186/s13104-018-3788-5 (PMC6161353; doi:10.1186/s13104-018-3788-5)
Supplement: Supplementary file 3 — Additional file 3. Confirmation results for guide RNA construction [file 13104_2018_3788_MOESM3_ESM.docx]

**Additional file 3: Sequencing results**

Guide construction was checked using FW primer 5’ CCTTTGAGTGAGCTGATACC 3' and RV primer 5'-CGAAGGCTTTAATTTGCGGC-3' to create 1000 bp plasmid fragments that were sequenced by sanger sequencing (Baseclear, The Netherlands).

**Sequencing results for p426_IAH1 guide RNA**

The incorporated gRNA sequence is displayed in green.

#=======================================

EMBOSS_001 1 GCTGATACCGCTCGCCGCAGCCGAACGACCGAGCGCAGCGAGTCAGTGAG 50

EMBOSS_001 51 CGAGGAAGCGGAAGAGCGCCCAATACGCAAACCGCCTCTCCCCGCGCGTT 100

EMBOSS_001 101 GGCCGATTCATTAATGCAGCTGGCACGACAGGTTTCCCGACTGGAAAGCG 150

EMBOSS_001 151 GGCAGTGAGCGCAACGCAATTAATGTGAGTTAGCTCACTCATTAGGCACC 200

EMBOSS_001 201 CCAGGCTTTACACTTTATGCTTCCGGCTCGTATGTTGTGTGGAATTGTGA 250

EMBOSS_001 251 GCGGATAACAATTTCACACAGGAAACAGCTATGACCATGATTACGCCAAG 300

EMBOSS_001 301 CGCGCAATTAACCCTCACTAAAGGGAACAAAAGCTGGAGCTTCTTTGAAA 350

EMBOSS_001 351 AGATAATGTATGATTATGCTTTCACTCATATTTATACAGAAACTTGATGT 400

EMBOSS_001 401 TTTCTTTCGAGTATATACAAGGTGATTACATGTACGTTTGAAGTACAACT 450

EMBOSS_001 451 CTAGATTTTGTAGTGCCCTCTTGGGCTAGCGGTAAAGGTGCGCATTTTTT 500

EMBOSS_001 501 CACACCCTACAATGTTCTGTTCAAAAGATTTTGGTCAAACGCTGTAGAAG 550

EMBOSS_001 551 TGAAAGTTGGTGCGCATGTTTCGGCGTTCGAAACTTCTCCGCAGTGAAAG 600

EMBOSS_001 601 ATAAATGATCATTATCGATAAATTCGGGGAGTTTTAGAGCTAGAAATAGC 650

EMBOSS_001 651 AAGTTAAAATAAGGCTAGTCCGTTATCAACTTGAAAAAGTGGCACCGAGT 700

EMBOSS_001 701 CGGTGGTGCTTTTTTTGTTTTTTATGTCTTCGAGTCATGTAATTAGTTAT 750

EMBOSS_001 751 GTCACGCTTACGTTCACGCCCTCCCCCCACATCCGCTCTAACCGAAAAGG 800

EMBOSS_001 801 AAGGAGTTAGACAACCTGAAGTCTAGGTCCCTATTTATTTTTTTATAGTT 850

EMBOSS_001 851 ATGTTAGTATTAAGAACGTTATTTATATTTCAAATTTTTCTTTTTTTTCT 900

EMBOSS_001 901 GTACAGACGCGTGTACGCATGTAACATTATACTGAAAACGTGC 944

**Sequencing results for TIP1 guide RNA**

Below the sequencing results for p426_TIP1 are displayed. The incorporated gRNA sequence is displayed in green.

EMBOSS_001 1 CCCTTGAGTGAGCTGATACCGCTCGCCGCAGCCGAACGACCGAGCGCAGC 50

EMBOSS_001 51 GAGTCAGTGAGCGAGGAAGNGGAAGAGCGCCCAATACGCAAACCGCCTCT 100

EMBOSS_001 101 CCCCGCGCGTTGGCCGATTCATTAATGCAGCTGGCACGACAGGTTTCCCG 150

EMBOSS_001 151 ACTGGAAAGCGGGCAGTGAGCGCAACGCAATTAATGTGAGTTAGCTCACT 200

EMBOSS_001 201 CATTAGGCACCCCAGGCTTTACACTTTATGCTTCCGGCTCGTATGTTGTG 250

EMBOSS_001 251 TGGAATTGTGAGCGGATAACAATTTCACACAGGAAACAGCTATGACCATG 300

EMBOSS_001 301 ATTACGCCAAGCGCGCAATTAACCCTCACTAAAGGGAACAAAAGCTGGAG 350

EMBOSS_001 351 CTTCTTTGAAAAGATAATGTATGATTATGCTTTCACTCATATTTATACAG 400

EMBOSS_001 401 AAACTTGATGTTTTCTTTCGAGTATATACAAGGTGATTACATGTACGTTT 450

EMBOSS_001 451 GAAGTACAACTCTAGATTTTGTAGTGCCCTCTTGGGCTAGCGGTAAAGGT 500

EMBOSS_001 501 GCGCATTTTTTCACACCCTACAATGTTCTGTTCAAAAGATTTTGGTCAAA 550

EMBOSS_001 551 CGCTGTAGAAGTGAAAGTTGGTGCGCATGTTTCGGCGTTCGAAACTTCTC 600

EMBOSS_001 601 CGCAGTGAAAGATAAATGATCGGAAGAAGCTGGGGAAACGGGTTTTAGAG 650

EMBOSS_001 651 CTAGAAATAGCAAGTTAAAATAAGGCTAGTCCGTTATCAACTTGAAAAAG 700

EMBOSS_001 701 TGGCACCGAGTCGGTGGTGCTTTTTTTGTTTTTTATGTCTTCGAGTCATG 750

EMBOSS_001 751 TAATTAGTTATGTCACGCTTACGTTCACGCCCTCCCCCCACATCCGCTCT 800

EMBOSS_001 801 AACCGAAAAGGAAGGAGTTAGACAACCTGAAGTCTAGGTCCCTATTTATT 850

EMBOSS_001 851 TTTTTATAGTTATGTTAGTATTAAGAACGTTATTTATATTTCAAATTTTT 900

EMBOSS_001 901 CTTTTTTTTCTGTACAGACGCGTGTACGCATGTAACATTATAC 943
