# Supplementary material for: CRISPR-Cas genome engineering of esterase activity in Saccharomyces cerevisiae steers aroma formation
Source: BMC Res Notes. 2018 Sep 27;11:682. doi: 10.1186/s13104-018-3788-5 (PMC6161353; doi:10.1186/s13104-018-3788-5)
Supplement: Supplementary file 4 — Additional file 4. DNA repair fragment sequences used in this study during transformations. [file 13104_2018_3788_MOESM4_ESM.docx]

**Additional file 4: Linear DNA repair fragments**

Gene knockouts were made by introduction of linear repair fragments. Homology arms upstream and downstream of the stop codon were designed for efficient homologous recombination repair. The left and right homology arms are separated by the start and stop codon of the original gene displayed in lowercase letters and annotated with pink and purple (atg and tga respectively).

The repair template sequence for the donor oligonucleotide for knocking out IAH1 is shown in Figure S1.


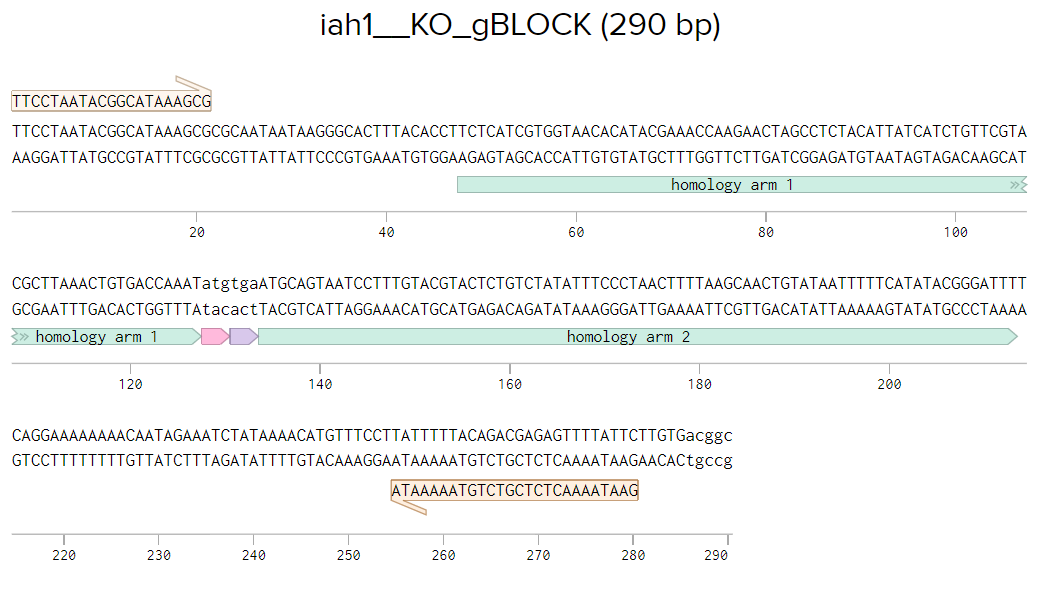


Figure S1: IAH1 knock out gBlock repair fragment (290bp length) and amplification primers

The repair template sequence for the donor oligonucleotide for knocking out TIP1 is shown in Figure S2. The left and right homology arms are separated by the start and stop codon of the original gene displayed in lowercase letters and annotated with orange and cyan (atg and taa respectively).


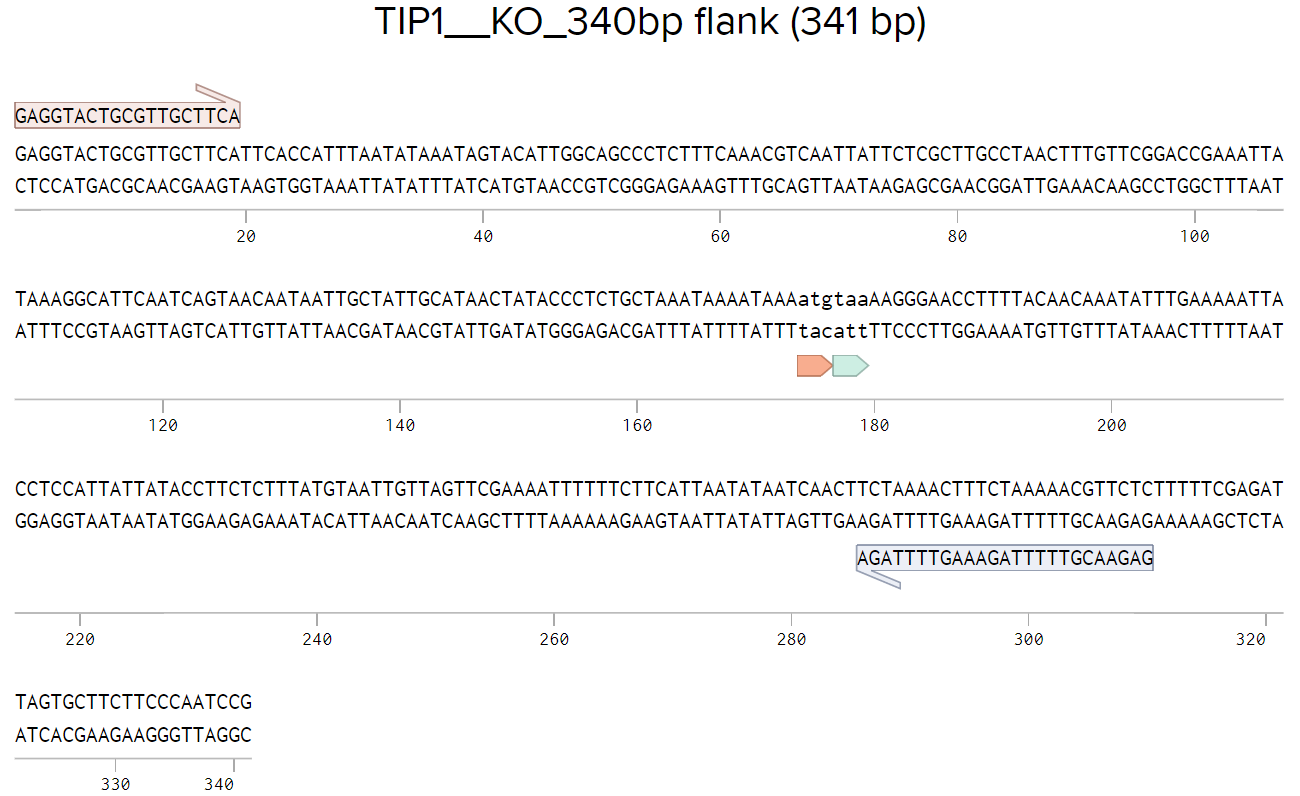


Figure S2: TIP1 knock out gBlock repair fragment (341 bp length) and amplification primers
