# Supplementary material for: CRISPR-Cas genome engineering of esterase activity in Saccharomyces cerevisiae steers aroma formation
Source: BMC Res Notes. 2018 Sep 27;11:682. doi: 10.1186/s13104-018-3788-5 (PMC6161353; doi:10.1186/s13104-018-3788-5)
Supplement: Supplementary file 5 — Additional file 5. Protocol used to transform linear repair fragment DNA and plasmids into S. cerevisiae [file 13104_2018_3788_MOESM5_ESM.docx]

**Additional file 5: Transformation protocol**

The following protocol transformation of plasmid p426-SNR52p-gRNA.TIP1/Y-SUP4t and p415-GaIL-TEF1-CAS9-CYC1t was used.

All centrifugations at 2250g for 3 minutes. Keep the cells on ice as much as possible during the protocol

1. Grow yeast in 10 ml Synthetic Complete (- auxotrophic requirements) at 30°C overnight
2. Inoculate fresh 10 ml SC(- auxotrophic requirements) to OD600=0.3, grow for 5 hours at 30°C (until OD600=1.8)
3. Pellet and wash with 10 ml ice-cold water
4. Pellet and wash with 10 ml ice-cold electroporation buffer (1 M Sorbitol/1 mM CaCl2)
5. Pellet and resuspend in 2 ml 500 mM LiAC/10 mM DTT
6. Incubate (shaker or roller drum) for 30 minutes at 30°C
7. Pellet and wash with 10 ml ice-cold electroporation buffer
8. Pellet and resuspend in 1.6 ml ice-cold electroporation buffer
9. Mix 400 µl cells with 1 nmol oligonucleotide (repair fragment), 1 µg plasmid cassette and 10 µl salmon DNA in an Eppendorf tube for each transformation.
10. Electroporate at 2.5 kV, 25 µF and 200Ω
11. Transfer electroporated cells to 7 ml 1:1 1M sorbitol/YPD medium as quickly as possible
12. Incubate (shaken) at 30°C overnight
13. Plate 100 µl 10^-5^ diluted cells on YPD and 50 µl and 200 µl undiluted on selective SC plates, spin down and also plate the pellet on a selective SC plates.
