# Supplementary material for: CRISPR-Cas genome engineering of esterase activity in Saccharomyces cerevisiae steers aroma formation
Source: BMC Res Notes. 2018 Sep 27;11:682. doi: 10.1186/s13104-018-3788-5 (PMC6161353; doi:10.1186/s13104-018-3788-5)

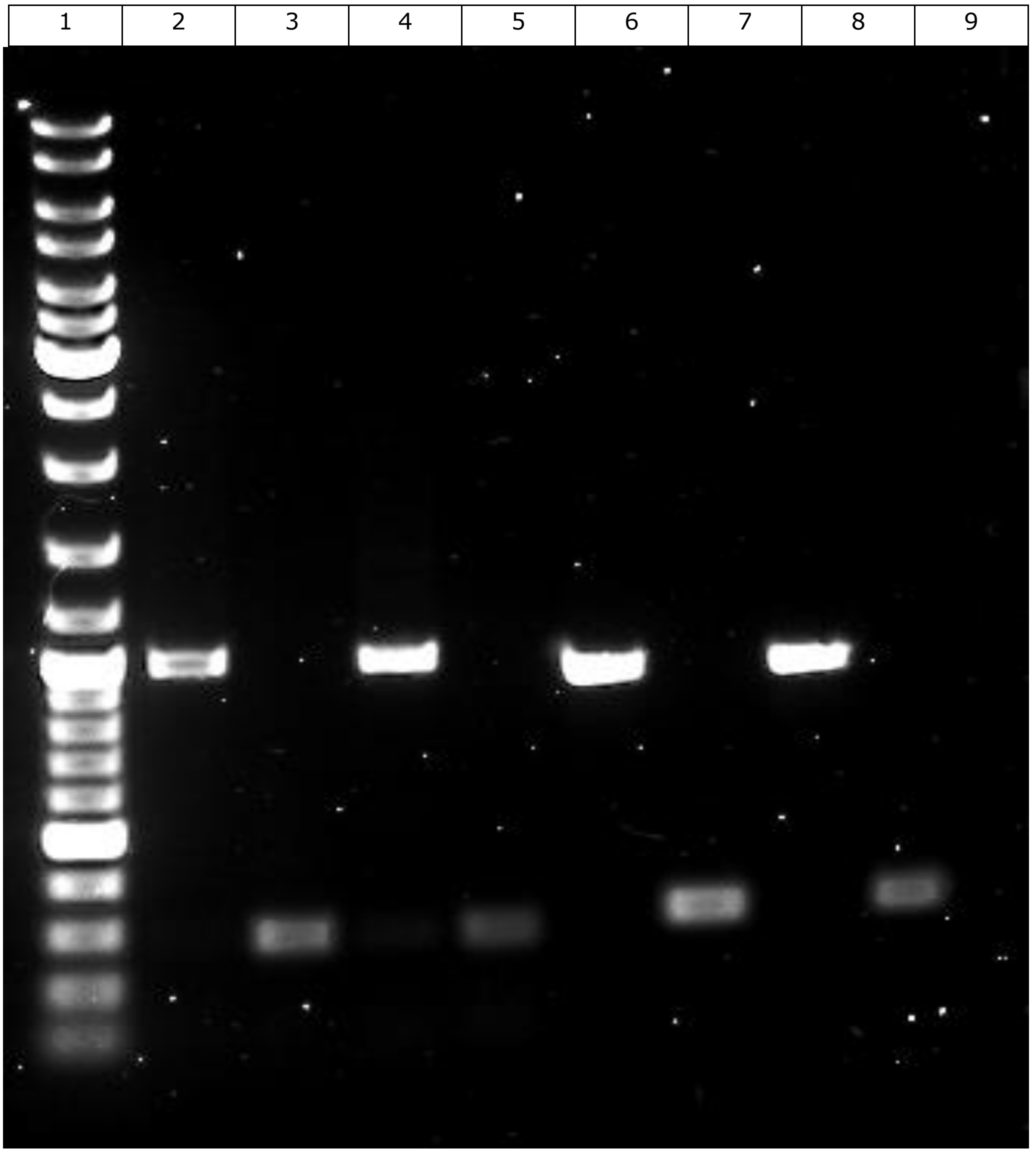

Figure S3: PCR gel of products send to sequencing. Products were created using primers that bind approximately 150 bp upstream and downstream of IAH1 and *TIP1* respectively. *1. Generuler 10kB DNA Ladder with highlights at 500 bp, 1 kB and 3 kB. 2. Wild type IAH1* gene, 3. *ΔIAH1* mutant *IAH1* gene, 4. *ΔTIP* mutant *IAH1* gene, 5. *ΔIAH1ΔTIP* mutant *IAH1* gene, 6. Wild type *TIP1* gene, 7. *ΔTIP1* mutant *TIP1* gene, 8. *ΔIAH11* mutant *TIP1* gene, 9. *ΔIAH1ΔTIP* mutant *TIP1* gene.

Sequencing showed intact genes for both *IAH1* and *TIP1* in the wild type. *IAH1* was found to be intact in *ΔTIP1*, *TIP1* was found to be intact in *ΔIAH1.* All deletions were found to be successful in removing the complete target gene excluding start and stop codon and without mutations closely upstream and downstream the target genes in all mutants. For clarity start and stop codons and homology arms upstream and downstream of the target genes are annotated in mutant sequencing results. Sequencing results are displayed on the following pages wherein d=Δ. i.e. d*IAH*1 *IAH1* is the result for sequencing the product for primers binding upstream and downstream of *IAH1* for the *ΔIAH1* mutant.

(from 1-856 bp)

Wild type IAH1 gene (862 bp)

AACACATACGAAACCAAGAACTAGCCTCTACATTATCATCTGTTCGTACGCTTAAACTGTGACCAAATATGGATTACGAGAAGTTTCTGKTATTTGGGGATTCCATT
TTGTGTATGCTTTGGTTCTTGATCGGAGATGTAATAGTAGACAAGCATGCGAATTTGACACTGGTTTATACCTAATGCTCTTCAAAGACMATAAACCCCTAAGGTAA

Homology arm 1 | IAH1

20 40 60 80 100

ACTGAATTTGCTTTTAATACTAGGCCCATTGAAGATGGCAAAGATCAGTATGCTCTTGGAGCCGCATTAGTCAACGAATATACGAGAAAAATGGATATTCTTCAAAG
TGACTTAAACGAAAATTATGATCCGGGTAACTTCTACCGTTTCTAGTCATACGAGAACCTCGGCGTAATCAGTTGCTTATATGCTCTTTTTACCTATAAGAAGTTTC

IAH1

120 140 160 180 200

AGGGTTCAAAGGGTACACTTCTAGATGGGCGTTGAAAATACTTCCTGAGATTTTAAAGCATGAATCCAATATTGTCATGGCCACAATATTTTTGGGTGCCAACGATG
TCCCAAGTTTCCCATGTGAAGATCTACCCGCAACTTTTATGAAGGACTCTAAAATTTCGTACTTAGGTTATAACAGTACCGGTGTTATAAAAACCCACGGTTGCTAC

IAH1

220 240 260 280 300 320

CATGCTCAGCAGGTCCCCAAAGTGTCCCCCTCCCCGAATTTATCGATAATATTCGTCAAATGGTATCTTTGATGAAGTCTTACCATATCCGTCCTATTATAATAGGA
GTACGAGTCGTCCAGGGGTTTCACAGGGGGAGGGGCTTAAATAGCTATTATAAGCAGTTTACCATAGAAACTACTTCAGAATGGTATAGGCAGGATAATATTATCCT

IAH1

340 360 380 400 420

CCGGGGCTAGTAGATAGAGAGAAGTGGGAAAAAGAAAAATCTGAAGAAATAGCTCTCGGATACTTCCGTACCAACGAGAACTTTGCCATTTATTCCGATGCCTTAGC
GGCCCCGATCATCTATCTCTCTTCACCCTTTTTCTTTTTAGACTTCTTTATCGAGAGCCTATGAAGGCATGGTTGCTCTTGAAACGGTAAATAAGGCTACGGAATCG

IAH1

440 460 480 500 520

AAAACTAGCCAATGAGGAAAAAGTTCCCTTCGTGGCTTTGAATAAGGCGTTTCAACAGGAAGGTGGTGATGCTTGGCAACAACTGCTAACAGATGGACTGCACTTTT
TTTTGATCGGTTACTCCTTTTTCAAGGGAAGCACCGAAACTTATTCCGCAAAGTTGTCCTTCCACCACTACGAACCGTTGTTGACGATTGTCTACCTGACGTGAAAA

IAH1

540 560 580 600 620 640

CCGGAAAAGGGTACAAAATTTTTCATGACGAATTATTGAAGGTCATTGAGACATTCTACCCCCAATATCATCCCAAAAACATGCAGTACAAACTGAAAGATTGGAGA
GGCCTTTTCCCATGTTTTAAAAAGTACTGCTTAATAACTTCCAGTAACTCTGTAAGATGGGGGTTATAGTAGGGTTTTTGTACGTCATGTTTGACTTTCTAACCTCT

IAH1

660 680 700 720 740

GATGTGCTAGATGATGGATCTAACATAATGTCTTGAATGCAGTAATCCTTTGTACGTACTCTGTCTATATTTCCCTAACTTTTAAGCAACTGTATAATTTTTCATAT
CTACACGATCTACTACCTAGATTGTATTACAGAACTTACGTCATTAGGAAACATGCATGAGACAGATATAAAGGGATTGAAAATTCGTTGACATATTAAAAAGTATA

IAH1 | Homology arm 2

760 780 800 820 840

Wild type IAH1 gene (862 bp) (from 857-862 bp)

```
ACGGGA
TGCCCT
```

860

Wild type TIP1 gene (767 bp)

ATCAGTAACAATAATTGCTATTGCATAACTATACCCTCTGCTAAATAAAATAAAATGTCCGTTTCCAAGATTGCTTTCGTTTTAAGTGCCATTGCCTCTTTGGCCGT
TAGTCATTGTTATTAACGATAACGTATTGATATGGGAGACGATTTATTTTATTTTACAGGCAAAGGTTCTAACGAAAGCAAAATTCACGGTAACGGAGAAACCGGCA

Homology arm 3 | TIP1 gene

20 40 60 80 100

CGCTGACACCAGCGCCGCCGAAACTGCTGAATTGCAAGCTATTATCGGTGACATCAACTCTCATCTTTCTGACTACTTGGGTCTAGAAACTGGCAACAGTGGATTCC
GCGACTGTGGTCGCGGCGGCTTTGACGACTTAACGTTCGATAATAGCCACTGTAGTTGAGAGTAGAAAGACTGATGAACCCAGATCTTTGACCGTTGTCACCTAAGG

TIP1 gene

120 140 160 180 200

AAATTCCATCTGATGTCTTGAGTGTGTATCAACAAGTCATGACTTACACCGATGACGCTTACACTACCTTGTTTAGTGAATTGGACTTTGATGCTATCACTAAGACA
TTTAAGGTAGACTACAGAACTCACACATAGTTGTTCAGTACTGAATGTGGCTACTGCGAATGTGATGGAACAAATCACTTAACCTGAAACTACGATAGTGATTCTGT

TIP1 gene

220 240 260 280 300 320

ATTGTTAAATTGCCATGGTACACCACAAGATTGAGTTCTGAAATCGCTGCTGCTCTTGCCTCCGTTTCCCCAGCTTCTTCCGAGGCTGCATCTTCTTCCGAGGCTGC
TAACAATTTAACGGTACCATGTGGTGTTCTAACTCAAGACTTTAGCGACGACGAGAACGGAGGCAAAGGGGTCGAAGAAGGCTCCGACGTAGAAGAAGGCTCCGACG

TIP1 gene

340 360 380 400 420

ATCTTCTTCCAAGGCTGCATCTTCTTCCGAAGCTACATCCTCTGCCGCTCCATCCTCTTCTGCTGCCCCATCTTCTTCTGCTGCCCCATCATCATCTGCCGAATCAT
TAGAAGAAGGTTCCGACGTAGAAGAAGGCTTCGATGTAGGAGACGGCGAGGTAGGAGAAGACGACGGGGTAGAAGAAGACGACGGGGTAGTAGTAGACGGCTTAGTA

TIP1 gene

440 460 480 500 520

CTTCTAAGGCCGTTTCTTCTTCTGTCGCTCCAACTACCTCTTCTGTCAGCACTTCTACAGTCGAAACTGCTTCCAATGCCGGTCAAAGAGTCAATGCAGGCGCTGCC
GAAGATTCCGGCAAAGAAGAAGACAGCGAGGTTGATGGAGAAGACAGTCGTGAAGATGTCAGCTTTGACGAAGGTTACGGCCAGTTTCTCAGTTACGTCCGCGACGG

TIP1 gene

540 560 580 600 620 640

TCTTTCGGTGCTGTTGTTGCAGGTGCAGCTGCTTTATTGTTATAAAAGGGAACCTTTTACAACAAATATTTGAAAAATTACCTCCATTATTATACCTTCTCTTTATG
AGAAAGCCACGACAACAACGTCCACGTCGACGAAATAACAATATTTTCCCTTGGAAAATGTTGTTTATAAACTTTTTAATGGAGGTAATAATATGGAAGAGAAATAC

TIP1 gene | Homology arm 4

660 680 700 720 740

TAATTGTTAGTTCGAAAA
ATTAACAATCAAGCTTTT

750 760

dIAH1 IAH1 gene (232 bp)

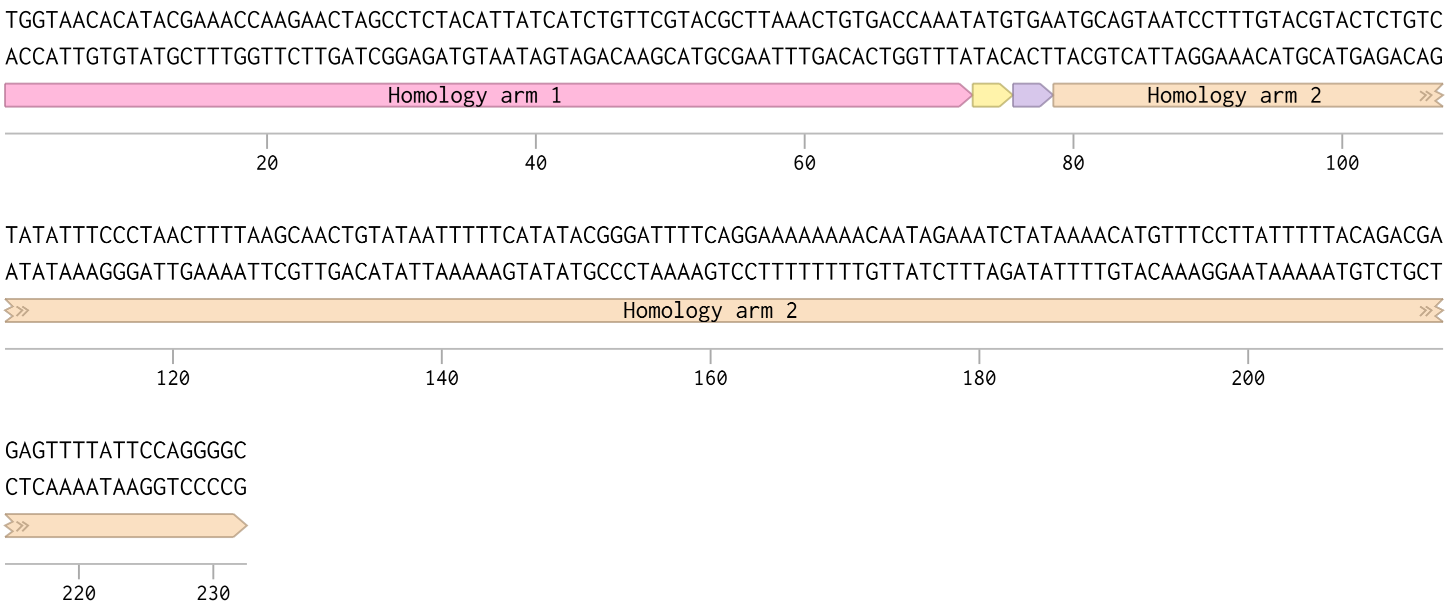

(from 1-856 bp)

dIAH1 TIP1 gene (896 bp)

TTGGCAGCCCTCTTTCAACGTCAATTATTCTCGCTTGCCTAACTTTGTTCGGACCGAAATTATAAAGGCATTCAATCAGTAACAATAATTGCTATTGCATAACTATA
AACCGTCGGGAGAAAGTTGCAGTTAATAAGAGCGAACGGATTGAAACAAGCCTGGCTTTAATATTTCCGTAAGTTAGTCATTGTTATTAACGATAACGTATTGATAT

Homology arm 3

20 40 60 80 100

CCCTCTGCTAAATAAAATAAAATGTCCGTTTCCAAGATTGCTTTCGTTTTAAGTGCCATTGCCTCTTTGGCCGTCGCTGACACCAGCGCCGCCGAAACTGCTGAATT
GGGAGACGATTTATTTTATTTTACAGGCAAAGGTTCTAACGAAAGCAAAATTCACGGTAACGGAGAAACCGGCAGCGACTGTGGTCGCGGCGGCTTTGACGACTTAA

Homology arm 3 | TIP1

120 140 160 180 200

GCAAGCTATTATCGGTGACATCAACTCTCATCTTTCTGACTACTTGGGTCTAGAAACTGGCAACAGTGGATTCCAAATTCCATCTGATGTCTTGAGTGTGTATCAAC
CGTTCGATAATAGCCACTGTAGTTGAGAGTAGAAAGACTGATGAACCCAGATCTTTGACCGTTGTCACCTAAGGTTTAAGGTAGACTACAGAACTCACACATAGTTG

TIP1

220 240 260 280 300 320

AAGTCATGACTTACACCGATGACGCTTACACTACCTTGTTTAGTGAATTGGACTTTGATGCTATCACTAAGACAATTGTTAAATTGCCATGGTACACCACAAGATTG
TTCAGTACTGAATGTGGCTACTGCGAATGTGATGGAACAAATCACTTAACCTGAAACTACGATAGTGATTCTGTTAACAATTTAACGGTACCATGTGGTGTTCTAAC

TIP1

340 360 380 400 420

AGTTCTGAAATCGCTGCTGCTCTTGCCTCCGTTTCCCCAGCTTCTTCCGAGGCTGCATCTTCTTCCGAGGCTGCATCTTCTTCCAAGGCTGCATCTTCTTCCGAAGC
TCAAGACTTTAGCGACGACGAGAACGGAGGCAAAGGGGTCGAAGAAGGCTCCGACGTAGAAGAAGGCTCCGACGTAGAAGAAGGTTCCGACGTAGAAGAAGGCTTCG

TIP1

440 460 480 500 520

TACATCCTCTGCCGCTCCATCCTCTTCTGCTGCCCCATCTTCTTCTGCTGCCCCATCATCATCTGCCGAATCATCTTCTAAGGCCGTTTCTTCTTCTGTCGCTCCAA
ATGTAGGAGACGGCGAGGTAGGAGAAGACGACGGGGTAGAAGAAGACGACGGGGTAGTAGTAGACGGCTTAGTAGAAGATTCCGGCAAAGAAGAAGACAGCGAGGTT

TIP1

540 560 580 600 620 640

CTACCTCTTCTGTCAGCACTTCTACAGTCGAAACTGCTTCCAATGCCGGTCAAAGAGTCAATGCAGGCGCTGCCTCTTTCGGTGCTGTTGTTGCAGGTGCAGCTGCT
GATGGAGAAGACAGTCGTGAAGATGTCAGCTTTGACGAAGGTTACGGCCAGTTTCTCAGTTACGTCCGCGACGGAGAAAGCCACGACAACAACGTCCACGTCGACGA

TIP1

660 680 700 720 740

TTATTGTTATAAAAGGGAACCTTTTACAACAAATATTTGAAAAATTACCTCCATTATTATACCTTCTCTTTATGTAATTGTTAGTTCGAAAATTTTTTCTTCATTAA
AATAACAATATTTTCCCTTGGAAAATGTTGTTTATAAACTTTTTAATGGAGGTAATAATATGGAAGAGAAATACATTAACAATCAAGCTTTTAAAAAAGAAGTAATT

TIP1 | Homology arm 4

760 780 800 820 840

dIAH1 TIP1 gene (896 bp) (from 857-896 bp)

TATAATCAACTTCTAAAACWTTCTAAAAACGTTCTACCCT
ATATTAGTTGAAGATTTTGWAAGATTTTTGCAAGATGGGA

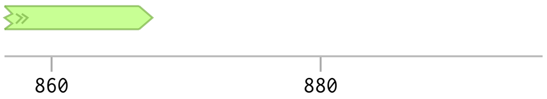

(from 1-856 bp)

dTIP1 IAH1 gene (871 bp)

TTCCTGAAAATCCCGTATATGAAAAATTATACAGTTGCTTAAAAGTTAGGGAAATATAGACAGAGTACGTACAAAGGATTACTGCATTCAAGACATTATGTTAGATC
AAGGACTTTTAGGGCATATACTTTTTAATATGTCAACGAATTTTCAATCCCTTTATATCTGTCTCATGCATGTTTCCTAATGACGTAAGTTCTGTAATACAATCTAG

Homology arm 2 IAH1

20 40 60 80 100

CATCATCTAGCACATCTCTCCAATCTTTCAGTTTGTACTGCATGTTTTTGGGATGATATTGGGGGTAGAATGTCTCAATGACCTTCAATAATTCGTCATGAAAAATT
GTAGTAGATCGTGTAGAGAGGTTAGAAAGTCAAACATGACGTACAAAAACCCTACTATAACCCCCATCTTACAGAGTTACTGGAAGTTATTAAGCAGTACTTTTTAA

IAH1

120 140 160 180 200

TTGTACCCTTTTCCGGAAAAGTGCAGTCCATCTGTTAGCAGTTGTTGCCAAGCATCACCACCTTCCTGTTGAAACGCCTTATTCAAAGCCACGAAGGGAACTTTTTC
AACATGGGAAAAGGCCTTTTCACGTCAGGTAGACAATCGTCAACAACGGTTCGTAGTGGTGGAAGGACAACTTTGCGGAATAAGTTTCGGTGCTTCCCTTGAAAAAG

IAH1

220 240 260 280 300 320

CTCATTGGCTAGTTTTGCTAAGGCATCGGAATAAATGGCAAAGTTCTCGTTGGTACGGAAGTATCCGAGAGCTATTTCTTCAGATTTTTCTTTTTCCCACTTCTCTC
GAGTAACCGATCAAAACGATTCCGTAGCCTTATTTACCGTTTCAAGAGCAACCATGCCTTCATAGGCTCTCGATAAAGAAGTCTAAAAAGAAAAAGGGTGAAGAGAG

IAH1

340 360 380 400 420

TATCTACTAGCCCCGGTCCTATTATAATAGGACGGATATGGTAAGACTTCATCAAAGATACCATTTGACGAATATTATCGATAAATTCGGGGAGGGGGACACTTTGG
ATAGATGATCGGGGCCAGGATAATATTATCCTGCCTATACCATTCTGAAGTAGTTTCTATGGTAAACTGCTTATAATAGCTATTTAAGCCCCTCCCCCTGTGAAACC

IAH1

440 460 480 500 520

GGACCTGCTGAGCATGCATCGTTGGCACCCAAAAATATTGTGGCCATGACAATATTGGATTCATGCTTTAAAATCTCAGGAAGTATTTTCAACGCCCATCTAGAAGT
CCTGGACGACTCGTACGTAGCAACCGTGGGTTTTTATAACACCGGTACTGTTATAACCTAAGTACGAAATTTTAGAGTCCTTCATAAAAGTTGCGGGTAGATCTTCA

IAH1

540 560 580 600 620 640

GTACCCTTTGAACCCTCTTTGAAGAATATCCATTTTTCTCGTATATTCGTTGACTAATGCGGCTCCAAGAGCATACTGATCTTTGCCATCTTCAATGGGCCTAGTAT
CATGGGAAACTTGGGAGAAACTTCTTATAGGTAAAAAGAGCATATAAGCAACTGATTACGCCGAGGTTCTCGTATGACTAGAAACGGTAGAAGTTACCCGGATCATA

IAH1

660 680 700 720 740

TAAAAGCAAATTCAGTAATGGAATCCCCAAATAACAGAAACTTCTCGTAATCCATATTTGGTCACAGTTTAAGCGTACGAACAGATGATAATGTAGAGGCTAGTTCT
ATTTTCGTTTAAGTCATTACCTTAGGGGTTTATTGTCTTTGAAGAGCATTAGGTATAAACCAGTGTCAAATTCGCATGCTTGTCTACTATTACATCTCCGATCAAGA

IAH1 Homology arm 1

760 780 800 820 840

dTIP1 IAH1 gene (871 bp) (from 857-871 bp)

TGGTTTCGTATGTGT
ACCAAAGCATACACA

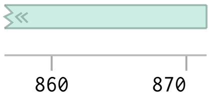

dTIP1 TIP1 gene (204 bp)

AACTAACAATTACATAAAGAGAAGGTATAATAATGGAGGTAATTTTTCAAATATTTGTTGTAAAAGGTTCCCTTTTACATTTTATTTTATTTAGCAGAGGGTATAGT
TTGATTGTTAATGTATTTCTCTTCCATATTATTACCTCCATTAAAAAGTTTATAAACAACATTTTCCAAGGGAAAATGTAAAATAAAATAAATCGTCTCCCATATCA

Homology arm 4 Homology arm 3

20 40 60 80 100

TATGCAATAGCAATTATTGTTACTGATTGAATGCCTTTATAATTTCGGTCCGAACAAAGTTAGGCAAGCGAGAATAATTGACGTTGAAAGAGGGCTG
ATACGTTATCGTTAATAACAATGACTAACTTACGGAAATATTAAAGCCAGGCTTGTTTCAATCCGTTCGCTCTTATTAACTGCAACTTTCTCCCGAC

Homology arm 3

110 120 130 140 150 160 170 180 190 200

dIAH1dTIP1 IAH1 gene (152 bp)

AAACCAAGAACTAGCCTCTACATTATCATCTGTTCGTACGCTTAAACTGKGACCAAATATGTGAATGCAGTAATCCTTTGTACGTACTCTGTCTATATTTCCCTAAC
TTTGGTTCTTGATCGGAGATGTAATAGTAGACAAGCATGCGAATTTGACMCTGGTTTATACACTTACGTCATTAGGAAACATGCATGAGACAGATATAAAGGGATTG

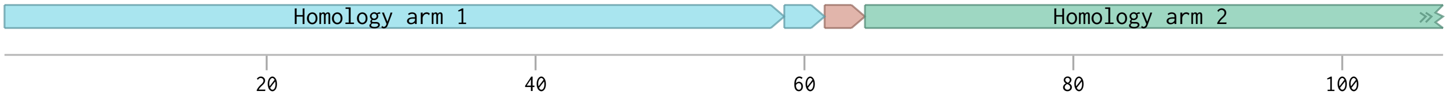

TTTTAAGCAACTGTATAATTTTTCATATACGGGATTTTCAGGAAA
AAAATTCGTTGACATATTAAAAAGTATATGCCCTAAAAGTCCTTT

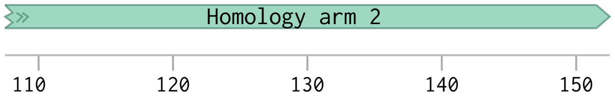

dIAH1dTIP1 TIP1 gene (87 bp)

TAATTTTTCAAATATTTGTTGTAAAAGGTTCCCTTTTACATTTTATTTTATTTAGCAGAGGGTATAGTTATGCAATAGCAATTATTG

ATTAAAAAGTTTATAAACAACATTTTCCAAGGGAAAATGTAAAATAAAATAAATCGTCTCCCATATCAATACGTTATCGTTAATAAC

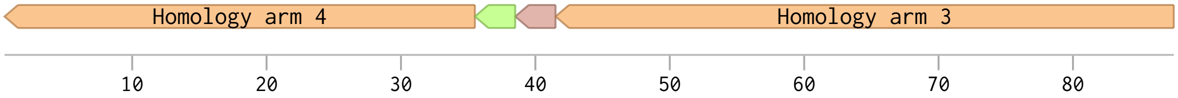

Supplement: Supplementary file 6 — Additional file 6. Sequencing result confirming gene deletion of IAH1 and TIP1 in mutant strains. [file 13104_2018_3788_MOESM6_ESM.pdf]
