# Supplementary material for: CRISPR-Cas genome engineering of esterase activity in Saccharomyces cerevisiae steers aroma formation
Source: BMC Res Notes. 2018 Sep 27;11:682. doi: 10.1186/s13104-018-3788-5 (PMC6161353; doi:10.1186/s13104-018-3788-5)
Supplement: Supplementary file 7 — Additional file 7. Protocols used to cultivate S. cerevisiae, to monitor esterase activity and to obtain aroma profiles. [file 13104_2018_3788_MOESM7_ESM.docx]

**Additional file 7: Methods by van Rijswijck et al. [1] used in this study.**

**Culture:**

1. Streak stock on MEA plate
2. Incubate overnight at 30°C or over the weekend at 20C
3. Inoculate 1 single colony in 10 ml MEB
4. Incubate overnight at 30°C 200RPM
5. Transfer 1.5 mL to an erlenmeyer containing 150 ml MEB
6. Incubate 48 hours at 30 °C static
7. Freeze 2 ml for HS SPME GC MS analysis

**Prepare cell extract:**

1. Measure OD600nm of the cultures
2. Spin down to obtain an OD600nm of 50 in 1 mL PBS for cFDA
3. Resuspend pellet in 1 ml PBS (pH 7.4)
4. Spin down max speed 5 min
5. Resuspend pellet in 700 μl PBS (pH 7.4)
6. Transfer to tube containing beads
7. Put on ice
8. Beadbeat 9* 20 sec with 1 min on ice in between
9. Spin down max speed 5 min at 4 °C
10. Combine supernatant of the same strain/condition

**Determine protein content:**

1. pipet 5 μl CE or standard in the appropriate wells
2. add 325 μl bradford reagent
3. mix and wait for 10 min at room temperature
4. measure absorbance at 595nm
5. calculate the protein concentration in μg/ml

**Esterase activity:**

1. pipet 40 μl CE in the appropriate wells
2. add 160 μl Mc Ilvaine buffer (pH 7.3) containing cFDA (6µl cFDA 0.2mM per ml Mc Ilvaine buffer to end concentration of 50 μM)
3. measure fluorescence at 494/515nm at 2 min intervals for 1 hour at 40°C

**Volatile organic compounds analysis:**

VOCs in each sample were determined by headspace solid-phase microextraction gas chromatography mass spectrometry (HS-SPME GC-MS) using a Trace 1300 Gas Chromatograph (Thermo Fisher) with a TriPlus RSH autosampler (Thermo Fisher) and an ISQ QD mass spectrometer (Thermo Fisher). Frozen samples were incubated at 60°C for 10 min. Volatile compounds were extracted for 20 min at 60°C using an SPME fibre (Car/DVB/PDMS, Supelco). The compounds were desorbed from the fibre for 2 min onto a Stabilwax®‐DA column (30 m length, 0.25 mm ID, 0.5 μm d_f_, Restek). The PTV was heated to 250 °C and operated in split mode at a ratio of 1:25. The GC oven temperature was kept at 40 °C for 2 min, raised to 240°C with a slope of 10°C/min and kept at 240°C for 5 min. Helium was used as carrier gas at a constant flow rate of 1.2 ml/min. Mass spectral data were collected over a range of m/z 33–250 in full‐scan mode with 3.0030 scans s^-1^.

Peak annotation was performed using Chromeleon® 7.2. NIST mainlib was used as reference compound library to match the mass spectra profiles with the profiles of NIST. Peak areas were calculated using MS quantitation peak area (highest m/z peak per compound). MS quantitation peak areas were corrected by fermentation performance (OD). Relative abundancy was calculated by the formula used by van Rijswijck et al. [1]:

$$Relative abundance \left( y \right) of compound \left( x \right)= \log_{2} (\frac{MSquantitation(xy)}{Median(MSquantitation \left( x \right))}$$

Heat maps were constructed using relative abundancy data in JMP 13 statistical software (SAS, USA).

Reference:

1. van Rijswijck IMH, Wolkers-Rooijackers JCM, Abee T, Smid EJ. Linking acetate ester hydrolysing activities to aroma profiles of *Cyberlindnera fabianii, Pichia kudriavzevii*and *Saccharomyces cerevisiae*. 2017. <https://library.wur.nl/WebQuery/wurpubs/fulltext/419524#page=45>.
